# Mitigating Groundwater Depletion in North China Plain with Cropping System that Alternate Deep and Shallow Rooted Crops

**DOI:** 10.3389/fpls.2017.00980

**Published:** 2017-06-08

**Authors:** Xiao-Lin Yang, Yuan-Quan Chen, Tammo S. Steenhuis, Steven Pacenka, Wang-Sheng Gao, Li Ma, Min Zhang, Peng Sui

**Affiliations:** ^1^College of Agronomy and Biotechnology, China Agricultural UniversityBeijing, China; ^2^Department of Biological and Environmental Engineering, Cornell University, IthacaNY, United States

**Keywords:** groundwater table decline, crop rotation, sequencing and vertical moisture complementarity, grain production, water management

## Abstract

In the North China Plain, groundwater tables have been dropping at unsustainable rates of 1 m per year due to irrigation of a double cropping system of winter wheat and summer maize. To reverse the trend, we examined whether alternative crop rotations could save water. Moisture contents were measured weekly at 20 cm intervals in the top 180 cm of soil as part of a 12-year field experiment with four crop rotations: sweet potato→ cotton→ sweet potato→ winter wheat-summer maize (SpCSpWS, 4-year cycle); peanuts → winter wheat-summer maize (PWS, 2-year cycle); ryegrass–cotton→ peanuts→ winter wheat-summer maize (RCPWS, 3-year cycle); and winter wheat-summer maize (WS, each year). We found that, compared to WS, the SpCSpWS annual evapotranspiration was 28% lower, PWS was 19% lower and RCPWS was 14% lower. The yield per unit of water evaporated improved for wheat within any alternative rotation compared to WS, increasing up to 19%. Average soil moisture contents at the sowing date of wheat in the SpCSpWS, PWS, and RCPWS rotations were 7, 4, and 10% higher than WS, respectively. The advantage of alternative rotations was that a deep rooted crop of winter wheat reaching down to 180 cm followed shallow rooted crops (sweet potato and peanut drawing soil moisture from 0 to 120 cm). They benefited from the sequencing and vertical complementarity of soil moisture extraction. Thus, replacing the traditional crop rotation with cropping system that involves rotating with annual shallow rooted crops is promising for reducing groundwater depletion in the North China Plain.

## Introduction

Groundwater – the world’s largest freshwater resource is essential to global food security by supporting irrigation of crops in periods without sufficient rainfall (Piao et al., 2010). Yet its depletion is widespread in both semi-arid and humid regions of the world (Aeschbach-Hertig and Gleeson, 2012). The aggregated groundwater depletion impacts on water resources are most obvious at the regional scale, such as in highly intensified agricultural parts of India, China, and the United States (Aeschbach-Hertig and Gleeson, 2012). Food production in such regions is only sustainable in the long term if groundwater levels are stabilized.

In changing from one rainfed crop to double cropping of winter wheat and summer maize with groundwater irrigation 40 years ago, grain production has increased greatly (Li et al., 2009). Currently, the winter wheat-summer maize double cropping system is the main cropping system in the North China Plain and accounts for 61% of the nation’s wheat and 45% of maize yields (National Bureau of Statistics of China, 2008). Annual precipitation fluctuates considerably around an average value of 500–600 mm, exhibiting a high variation of 300–1000 mm (Cao et al., 2014). More than 70% of the annual rainfall is concentrated from July to September, the summer maize growing season. Precipitation during the full growing season of winter wheat can only meet roughly 25–40% of the water needed for an optimal yield (Yang et al., 2015a,b). Therefore, more than 60% of the water needed for winter wheat must be pumped from the groundwater to maintain a consistently high yield level under the variable climate conditions. There is not sufficient annual recharge to replace this much withdrawn groundwater (Li et al., 2009). Pumping has resulted in about a 1.0–1.5 m per year decline in the groundwater table over the whole North China Plain over the past 20 years (Kendy et al., 2003; Fang et al., 2010; Yuan and Shen, 2013; Yang et al., 2015b). Consequently, a 50,000 km^2^ cone of depression in the groundwater table, the largest recorded worldwide, has formed in this area (Hu et al., 2005). Meanwhile, depletion of the region’s groundwater resources and pollution of surface and groundwater bodies have caused serious environmental and ecological problems (Liu et al., 2001). Without the change and adjustment of the conventional winter wheat-summer maize cropping system, the groundwater table will continue to fall, even though many agricultural water-saving technologies have been implemented during the past 20 years. Therefore, other water-saving practices should be developed, that maintain high crop production and improve water use efficiency. One of these practices on which this paper focuses is alternative crop rotations having a lower water demand. We are especially interested in exploring how crop sequencing within alternative crop rotations influences water use and offers opportunities for control.

Research in the North China Plain, including field experiments (Zhang et al., 2004, 2011; Sun et al., 2006, 2010; Du et al., 2010; Kong et al., 2012; Liu et al., 2013) and modeling studies (Wang et al., 2001; Kendy et al., 2003; Yang et al., 2006; Yu et al., 2006; Chen et al., 2010; Ma et al., 2013) revealed that the actual evapotranspiration of the winter wheat-summer maize system ranged from 610 to 870 mm. Due to the discrepancy between rainfall and water demand of the double cropping system, previous studies concluded that any attempts to meet the crop water deficit by irrigation with groundwater will result in a continuing decline of the groundwater table (Kendy et al., 2004; Hu et al., 2005; Sun et al., 2011; Meng et al., 2012; Yang et al., 2015b). Considering that the water consumption of the WS cropping system is not sustainable, and the more and more serious accumulating water deficit in this region, alternative cropping systems with lower water demands should be encouraged (Liao and Huang, 2004; Fang et al., 2010; Yang et al., 2015a,b). Diversifying cropping systems by including peanut, sweet potato, cotton, spring maize, or spring soybean as the preceding crop to the winter wheat-summer maize rotation (three crops in 2 years), has demonstrated the ability to lower annual average water consumption, provide good water use efficiency, and maintain the food production needed from this vital agricultural zone (Liu et al., 2008a,b; Ye et al., 2008; Fang et al., 2010; Sun et al., 2011; Zhang et al., 2011; Yang et al., 2015b). However, none of these studies looked in detail what mechanism caused the water-saving, thus confidence may be insufficient to implement changes. We will explore why sequencing certain crops saves water.

Previous research in the North China Plain mentioned above mostly focused on evaluation of the annual average water consumption of different crop rotations, or on design of the irrigation regime during the winter wheat growing season when water deficits generally occur. Research on the water saving mechanisms of alternative crop rotations and the influence of the sequencing within rotations is rarely reported. Under water-limited conditions, adequate initial soil moisture at the sowing stage is essential for crop germination, emergence, and plant establishment (Zhang et al., 2004). The soil moisture status at planting of a crop is affected substantially by the amount and distribution of precipitation and water use by the preceding crop, since there is essentially no fallow period between crops (Fang et al., 2010; Nielsen et al., 2015). The authors’ previous published paper (Yang et al., 2015b), concluded that diversified crop rotations could significantly decrease water consumption, which would potentially slow the groundwater table decline and markedly improve the economic water use efficiency. However, the actual water-saving mechanisms, such as the vertical soil water distribution, the *ET* variation and the irrigation across the previous and succeeding crops were not yet examined. It is currently not known how seasonal precipitation, irrigation (including amount and timing), and carryover effects of the earlier crops affect the soil water balance, crop yield, and *WUE* of winter wheat.

Therefore, this study explored the influences of soil moisture distribution, soil “reservoir” capacity, irrigation regime and precipitation variation of a preceding crop, such as sweet potato and peanut, on the succeeding winter wheat. The effects were quantified for different crop rotations using long sequences of plot -specific soil moisture profile measurements made at weekly intervals from October 2002 to October 2014. The objectives of this study were to expose and quantify the water-saving potential and mechanism of alternative crop rotations that include different annual crops before winter wheat and how this might mitigate the groundwater depletion. It will provide insight into how to adjust the planting structure to take control of the declining groundwater table in the North China Plain.

## Materials and Methods

### Experiment Site

**FIGURE 1:**
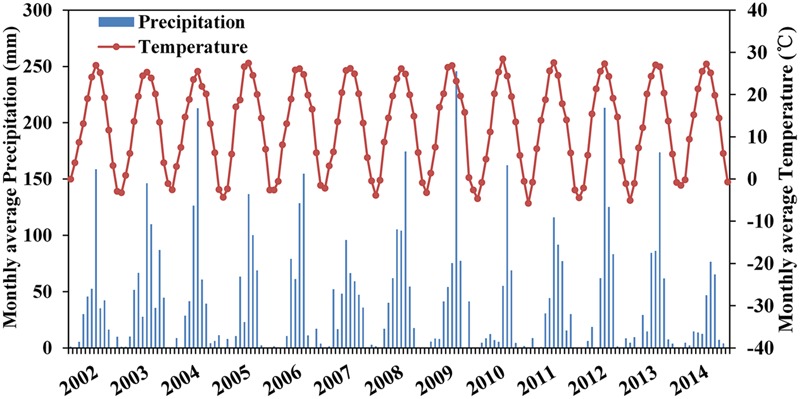
Monthly precipitation and temperature from 2002 to 2014 at Luancheng experiment site in the North China Plain.

The experiment was conducted in the field at the Luancheng Agro-Ecosystem Experimental Station, located in Luancheng County in Hebei Province in the northern part of the North China Plain at an elevation of 50.1 m (37°50′ N, 114°40′ E). The site is typical for agriculture in the northern part of North China Plain via its representative soil, water and field management practices. It has a warm, temperate zone, semi-humid, monsoon climate. The annual average temperature is 12.2°C with a monthly averages ranging from 26.3°C in July to 3.9°C in January. The average annual rainfall is 480 mm with 60–70% falling in the summer maize growing season from June to September. The monthly rainfall and average air temperature during the period of October, 2002- October, 2014 are displayed in **Figure 1**. The detailed soil type and soil characteristics including pH, total N, Olsen P, available K, organic matter, and hydraulic parameters including field capacity, wilting point, saturation, saturated hydraulic conductivity and unsaturated conductivity parameter (α^b^) were shown in the authors’ previous study (Yang et al., 2015b).

### Experiment Design and Crop Management

**FIGURE 2:**
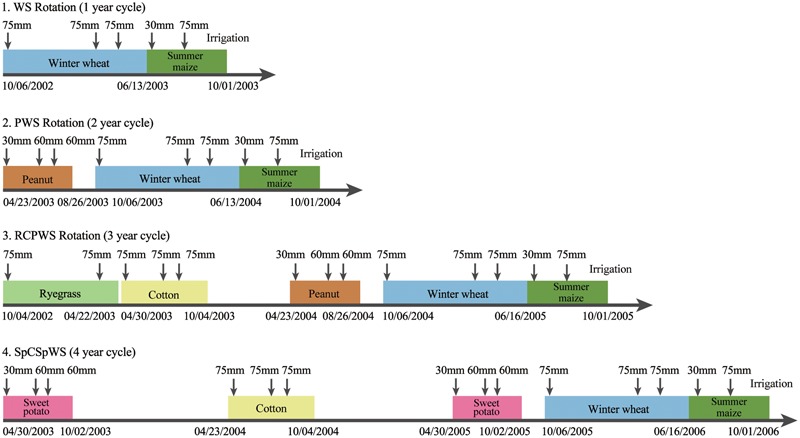
The crops grown and times and amount of irrigation water applied for the four crop rotations [Winter wheat (W) was irrigated at sowing, jointing and filling stages with a total of 225 mm; summer maize (S) at jointing and filling stages with a total of 105 mm; sweet potato (Sp) at sowing and middle stages with a total of 150 mm; cotton (C) at sowing, squaring and boll opening stages with a total of 225 mm; peanut (P) at sowing, flowering and pod forming stages with a total of 150 mm; ryegrass (R) at sowing and turning green stages with a total of 150 mm. SpCSpWS rotation, sweet potato→ cotton→ sweet potato → winter wheat-summer maize; RCPWS, ryegrass-cotton→ peanut → winter wheat-summer maize; PWS, peanut→ winter wheat-summer maize; WS, winter wheat-summer maize].

This crop rotation field experiment began in October 2002 and data were collected until October 2014. This experiment adopted a randomized complete-block design, with each treatment having three replicate plots of 30 m^2^ (4 m × 7.5 m). The plots were separated by a 1-m buffer zone without irrigation to minimize any interactions between adjacent plots. The experiment covered four cropping systems including (1) winter wheat-summer maize (symbolized by WS; 1-year cycle), (2) peanut → winter wheat-summer maize (PWS; 2-year cycle), (3) ryegrass–cotton→ peanuts→ winter wheat-summer maize (RCPWS; 3-year cycle), (4) sweet potato→ cotton→ sweet potato→ winter wheat-summer maize (S_P_CS_P_WS; 4-year cycle). The crop sequence and irrigation management of each crop in the four crop rotations, within their first rotation cycle, are shown in **Figure 2**. (All cycles then repeated to fill out 12 years.) A given crop within any rotation received the same agricultural management. The amounts of N, P, and K fertilizers followed typical local farming practices. The basal N, in form of urea, was applied by broadcasting before preparing the seedbed, and the topdressing was carried out in the crop growth season (Supplementary Table S1). Irrigation timings and amounts matched recommendations to regional farmers and were keyed to critical crop growth stages. Irrigation was fixed and not based on soil moisture or recent precipitation; however any “excess” irrigation water above evapotranspiration is not necessarily “lost” since it will percolate downward in this topographically flat area.

### Data Collection and Measurements

Daily weather data, including temperature, precipitation, and pan evaporation were collected from October 2002 to October 2014 using an automatic weather station 100 m away from the experiment site. Soil volumetric water content was monitored regularly, every 7 days during all the growing seasons, using a neutron probe (L520) with an interval depth of 20 cm from the soil surface to a depth of 2 m. Top 20 cm soil moisture was measured gravimetrically by oven drying soil cores to make up for the deficiency of using a neutron probe near the surface. Grain yield of each crop was measured after each harvest stage using the conventional method.

The annual and seasonal actual evapotranspiration of each crop and each cropping system were calculated based on a soil water balance model (Kendy et al., 2003, 2004). This model was validated for this site and shown to give good predictions of *ET*_a_ and percolation based on soil moisture content variation over time. The authors’ previous study details the calibration and validation of this model (Yang et al., 2015b). Individual water use efficiencies (*units kg yield per m^3^ ET_a_ water*) of winter wheat and summer maize were calculated as grain yield divided by seasonal *ET_a_*.

### Statistical Analysis

Analysis of variance (ANOVA) with Statistical Analysis System 9.3 (SAS Institute Inc., 2011) software helped test the significance of differences among the *ET_a_* of each rotation. ANOVA was also used to test the variation in soil moisture content within each soil profile, at different time points and with different treatments. A least significant difference (LSD) test at a 5% probability level determined any significant differences.

## Results

### Annual Average Water Consumption of Different Crop Rotations

**FIGURE 3:**
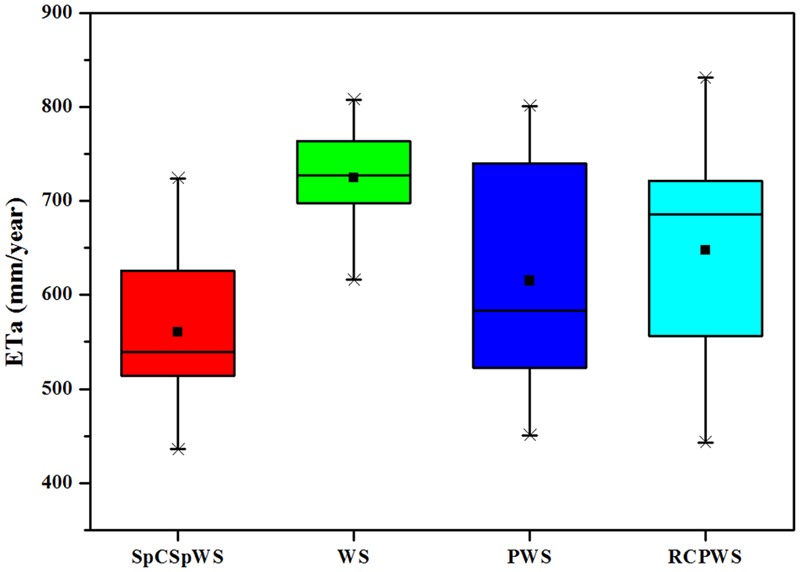
Annual actual evapotranspiration of four crop rotations from 2003 to 2014 at Luancheng site in the North China Plain. The boxplots represent the 25, 50, 75 percentiles. The squares and lines in the box plots indicate the mean and median, respectively. The crosses indicate the minimum and maximum. SpCSpWS rotation, sweet potato→ cotton→ sweet potato → winter wheat-summer maize; RCPWS, ryegrass-cotton→ peanut→ winter wheat-summer maize; PWS, peanut→ winter wheat-summer maize; WS, winter wheat-summer maize.

The annual average actual evapotranspiration amounts of different crop rotations during the period of 2003–2014 are shown in **Figure 3**. This is the same trend as seen in a previous study (Yang et al., 2015b) with a 12th year data added. Via ANOVA, the annual average *ET_a_* of the WS rotation, 725 mm, was significantly greater than that of other crop rotations (*P* < 0.05). The annual average *ET_a_* of sweet potato, cotton and peanuts were 465, 571, and 406 mm, respectively, which was markedly lower than that of the winter wheat-summer maize year (Supplementary Table S2). The SpCSpWS rotation had the smallest annual average *ET_a_* at 561 mm, 23% lower than the annual average *ET_a_* of the WS rotation. The annual average *ET_a_* of the PWS and RCPWS rotations were 615 and 647 mm, respectively, which saved 15 and 11% of water consumption compared to WS rotation. Each of the three alternative crop rotations saved water in the North China Plain, demonstrating their viabilities to improve the balance between food production and groundwater decline — providing that yields are not sacrificed too much to gain the water saving.

### Effects of Crop Rotation on the Yield and WUE of Winter Wheat in Different Crop Rotations

**FIGURE 4:**
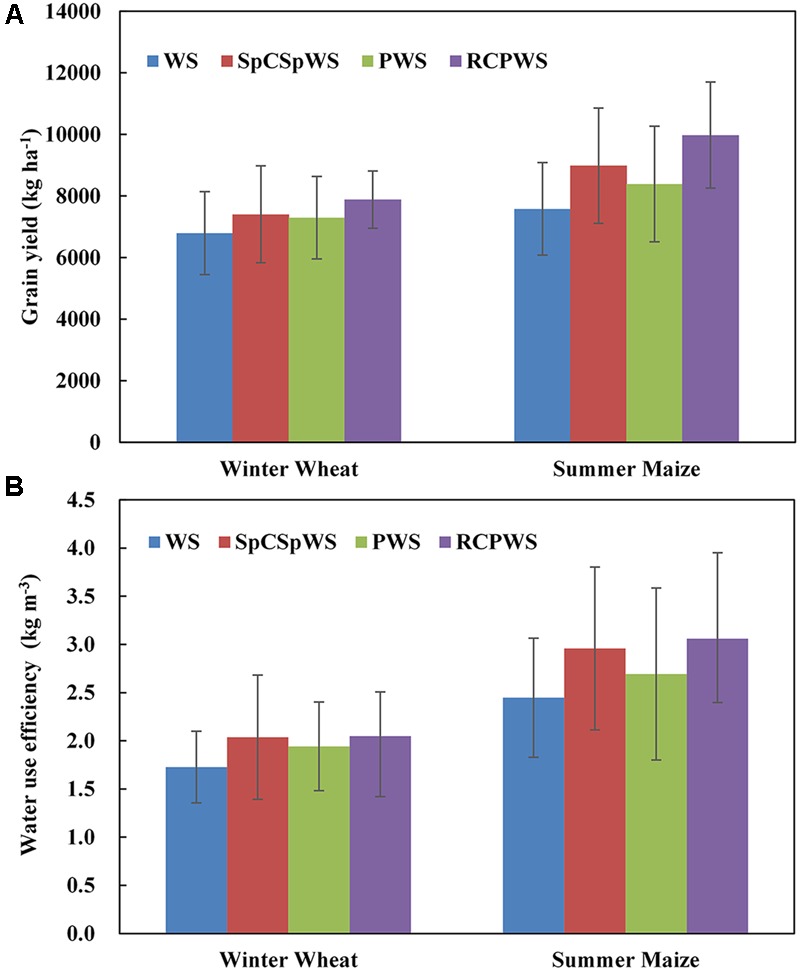
Annual average yield **(A)** and water use efficiency **(B)** of winter wheat and summer maize in the different crop rotations from 2003 to 2014. Bars represent the standard deviation of the replications; SpCSpWS rotation, sweet potato→ cotton → sweet potato → winter wheat-summer maize; RCPWS, ryegrass-cotton→ peanut→ winter wheat-summer maize; PWS, peanut→ winter wheat-summer maize; WS, winter wheat-summer maize.

The annual average yield of winter wheat and summer maize in the experiment’s different crop rotations from 2003 to 2014 are compared in **Figure 4A**. The annual average yield of winter wheat and summer maize in the WS rotation was the lowest at 6793 and 7582 kg ha^-1^, respectively. The annual average yield of winter wheat in the alternative crop rotations increased to a different degrees. A paired *t*-test of winter wheat yields in the alternative rotations compared to the WS rotation showed that wheat yields were significantly better in the SpCSpWS and PWS rotations (*P* < 0.05) and that wheat yields were insignificantly better in the RCPWS rotation (*P* = 0.11). The annual average yields of winter wheat in the SpCSpWS, PWS and RCPWS rotations are were 7406, 7294, and 7883 kg ha^-1^, or 9, 7, and 16% higher, respectively, than the annual average yield of winter wheat in the WS rotation. Similarly, the annual average yields of summer maize in the SpCSpWS, PWS, and RCPWS rotations were 8985, 8392, and 9978 kg ha^-1^, or 19, 11, and 32% higher, respectively, than the annual average yield of summer maize in the WS rotation. When yields of both winter wheat and maize are summed and compared pairwise, all three alternative rotations had better yield than WS (*P* < 0.04). Evidently, the benefits to wheat from previous conditions under the alternative preceding crop can also influence a following summer maize. Therefore, the diversified crop rotations which introduced alternative preceding crops, such as peanuts, cotton and sweet potato, supplied good soil water conditions so that the succeeding crops of winter wheat and summer maize could provide greater yield.

Further, the annual averages of *WUE* of both winter wheat and summer maize in the alternative crop rotations are much greater than those in the WS rotation from 2003 to 2014, despite lacking strong statistical significance (**Figure 4B**). The *WUE* of winter wheat in the SpCSpWS, PWS, and RCPWS rotations were 2.04, 1.94, and 2.05 kg m^-3^, whichwere18, 12, and 19% greater than the annual average *WUE*, 1.73 kg m^-3^, of winter wheat in the WS rotation, respectively. A paired *t*-test for *WUE* of winter wheat had similar results to the yields test. SpCSpWS and PWS had significantly better *WUE* (*P* < 0.05) and RCPWS an insignificantly better *WUE* (*P =* 0.22). Similarly, the annual average *WUE* of summer maize in the SpCSpWS, PWS and RCPWS rotations were 2.96, 2.63, and 3.06 kg m^-3^, or 21, 10, and 25% higher than that of the WS rotation, at 2.45 kg m^-3^, respectively. Therefore, the annual crops, which acted as the wheat-preceding crops in alternative crop rotations, improved the *WUE* of the succeeding crop of winter wheat, followed by summer maize.

### Carryover Soil Moisture between Crops in Different Crop Rotations

**FIGURE 5:**
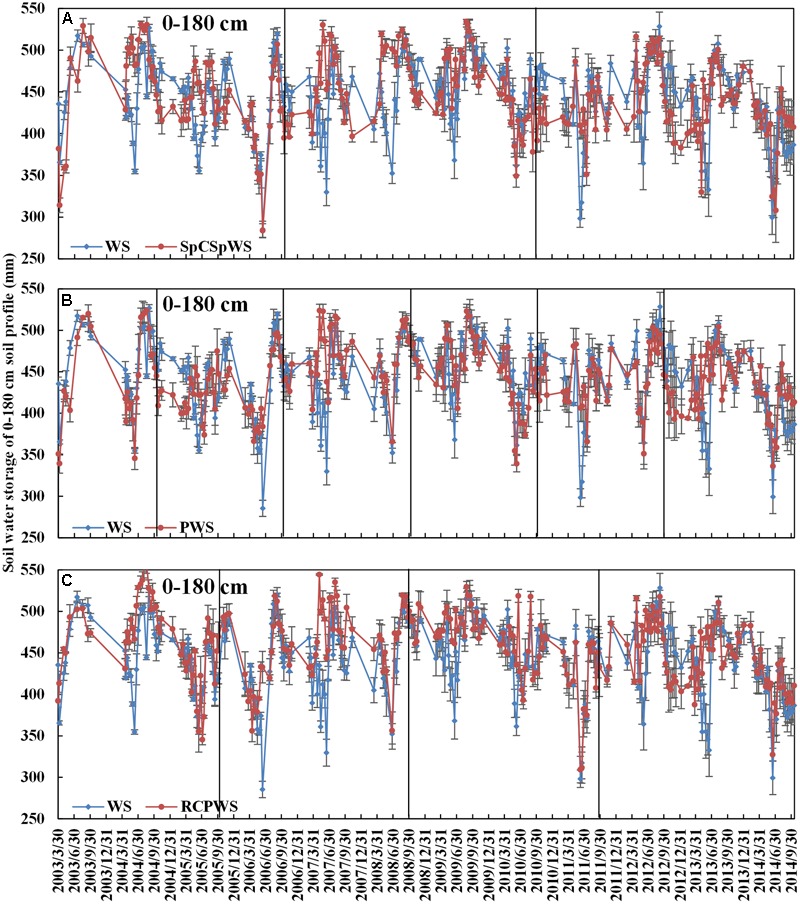
Water stored in the top 0–180 cm from 2003 to 2014 of the WS rotation with SpCSpWS rotation **(A)**, PWS rotation **(B)**, and RCPWS rotation **(C)**. Vertical black line indicates the start of new rotation. SpCSpWS rotation, sweet potato→ cotton→ sweet potato → winter wheat-summer maize; RCPWS, ryegrass-cotton→ peanut→ winter wheat-summer maize; PWS, peanut→ winter wheat-summer maize; WS, winter wheat-summer maize. Error bars display the means ± SD (*n* = 3).

**In the SpCSpWS rotation**, which involved a livestock crop (sweet potato), cash crop (cotton) and grain crop (winter wheat and summer maize) within a 4 year rotation cycle, the temporal variation of soil water storage of a 0–180 cm soil depth had clear differences compared to the WS rotation during the period of 2003–2014 (**Figure 5A**). The three points of lowest soil water storage in the SpCSpWS rotation occurred in three rotation cycles at the respective harvest dates of winter wheat in 2006, 2010, and 2014. For the WS rotation, the lowest soil water storage occurred at each annual harvest stage of winter wheat. Within the SpCSpWS rotation, the temporal variations in the range of soil moisture in cotton’s year with total irrigation of 225 mm and in sweet potato’s year with total irrigation of 150 mm were both lower than in the WS rotation even with its irrigation of 225 mm in the winter. The average soil water storage within a 0–180 cm soil depth from the beginning to the sowing date of winter wheat in the SpCSpWS rotation, across three rotation cycles from 2003 to 2014, increased by 62 mm. In contrast, soil moisture fell by 8 mm in the WS rotation for the same period (Supplementary Table S3). More information about the soil water storage changes within the 0–180 cm soil depth in each SpCSpWS rotation cycle is detailed in Supplementary Table S3. The average soil water storage of the 0–180 cm soil profile at the sowing date of winter wheat in SpSCpWS rotation, across three rotation cycles from 2003 to 2014, was 7% higher, at 463 mm, than in the WS rotation (**Figure 5A**). To sum up, compared to the WS continuous rotation, the SpCSpWS rotation (cotton and sweet potato preceding winter wheat and summer maize) increased the soil water storage at the beginning of the fourth part of the cycle, and thus provided a favorable soil moisture condition for winter wheat sowing. This could reduce the customary irrigation need for sowing winter wheat. Therefore, it appeared valuable to introduce an annual crop other than maize, during the high precipitation summer growing season, into the conventional WS cropping system, to effectively maintain a favorable later soil water status.

**In the PWS rotation**, which involved an oil crop (peanut) and grain crops (winter wheat and summer maize) within a 2 year rotation cycle, the lowest soil moisture content points occurred at the harvest dates of winter wheat in each rotation cycle, including 2004, 2006, 2008, 2010, 2012, and 2014 (**Figure 5B**). The soil water content from the harvest date of summer maize to the sowing date of the succeeding peanut crop was lower than that in the WS rotation, mainly because of non-productive evaporation from the bare soil in the fallow period from the middle of June to the following May. Concurrently, winter wheat in the WS rotation received irrigation pumped from the groundwater, which led to relatively high soil water storage (**Figure 5B**). The average soil water storage in the 0–180 cm soil depth from the beginning to the sowing date of winter wheat in the PWS rotation, across six rotation cycles, increased by 33 mm. Meanwhile, moisture dropped by an average of 15 mm in the WS rotation over the same period (Supplementary Table S4). More information about the soil water storage changes of the 0–180 cm soil depth in each PWS rotation cycle is detailed in Supplementary Table S4. The 471 mm average soil water storage in the 0–180 cm soil profile at the sowing date of winter wheat, in the PWS rotation across six rotation cycles, was 4% higher than in the WS rotation (**Figure 5B**). Therefore, the PWS rotation involving peanut crops, with high rainfall in the growing season, could provide good soil water conditions for the succeeding crop and potentially reduce its irrigation requirement.

**In the RCPWS rotation**, which included ryegrass as the first winter catch crop, a cash crop (cotton), an oil crop (peanuts) and grain crops (winter wheat and summer maize) within a 3 year rotation cycle, the lowest soil water storage contents of the 0–180 cm soil depth occurred at each harvest stage of winter wheat (including 2005, 2008, 2011, and 2014) (**Figure 5C**). In the ryegrass growth season in each RCPWS rotation cycle from 2003 to 2014, the soil moisture storage of the 0–180 cm soil depth was similar to that of the winter wheat growing season in the WS rotation. The similarities were mainly due to their shared physiological moisture property and ecological characteristics of winter wheat. Ryegrass has a shorter growing period and a lower irrigation requirement of just 150 mm compared to that of winter wheat of 225 mm. In the fallow period after the harvest of cotton and before the sowing of peanut in each rotation cycle, the soil moisture storage content of the 0–180 cm soil depth in the RCPWS rotation was lower than that in WS rotation. The main reason for this was the non-productive soil evaporation from the bare surface in the dry winter and the irrigation of WS in the winter (**Figure 5C**). The average soil water storage of the 0–180 cm soil depth from the beginning to the sowing date of winter wheat, in all four RCPWS rotation cycles from 2003 to 2014 increased by 33 mm, compared to 7 mm in the WS rotation (Supplementary Table S5). More information about the soil water storage changes of the 0–180 cm soil depth in each RCPWS rotation cycle is detailed in Supplementary Table S5. The average soil water storage of the 0–180 cm soil profile at the sowing date of winter wheat across the PWS six rotation cycles, from 2003 to 2014, was 479 mm, 10% higher than that in the WS rotation (**Figure 5C**). In summary, the preceding peanut crop provided a favorable soil moisture condition for sowing the succeeding winter wheat, which could have reduced the irrigation demand for sowing winter wheat, thus reducing the groundwater pumping for irrigation in the wheat-growing season.

### Profile Complementarity of Soil Moisture in Different Crop Rotations

**FIGURE 6:**
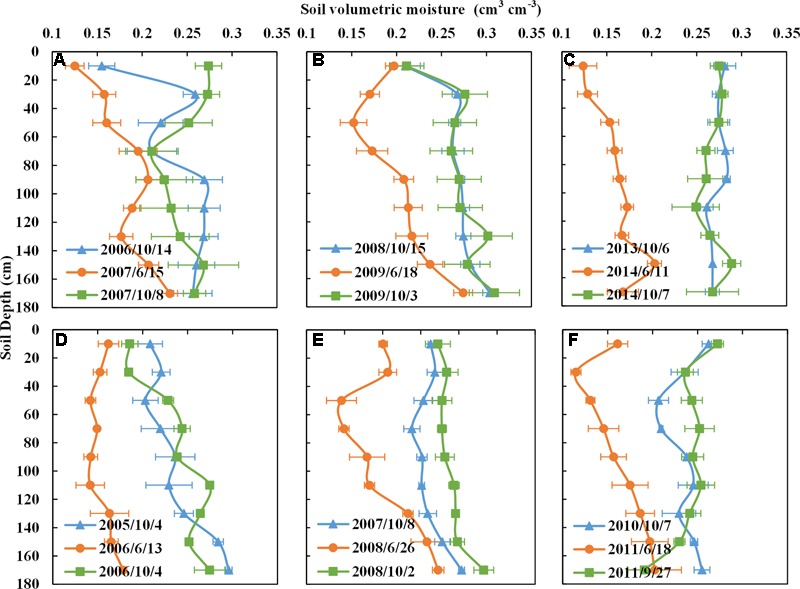
Soil moisure contents with depth of winter wheat-summer maize in different rotations for selected years. **(A–C)** Come from WS rotation; **(D–F)** from SpCSpWS, PWS, and RCPWS rotations, respectively. Blue line represents the soil miosture content with depth at the sowing date of winter wheat; orange line is the moisture content at the harvest date of winter wheat, which is also the sowing date of summer maize, green line is the mositure content at the harvest date of summer maize. WS, winter wheat-summer maize; SpCSpWS rotation, sweet potato→ cotton→ sweet potato → winter wheat-summer maize; RCPWS, ryegrass-cotton →peanut→ winter wheat-summer maize; PWS, peanut→ winter wheat-summer maize.

**In the WS rotation**, there was a significant decrease of soil moisture within the 0–180 cm soil profile from the sowing date (blue line) to the harvest date (orange line) of winter wheat (**Figures 6A–C**) (Detailed soil moisture profiles at sowing of winter wheat, harvesting of winter wheat or sowing of summer maize and at harvesting of summer maize are shown in Supplementary Figure S1A). The respective profiles showed that winter wheat could use soil water from the surface down to a depth of 180 cm. Due to the mismatch between the water requirement in the winter wheat growing season and precipitation, a large amount of groundwater was needed for irrigation. The soil moisture at harvest (orange curve) was evidently lower than that at sowing (blue curve) due to the high water consumption in the growing seasons from 2003 to 2014 (Supplementary Figure S1A). After winter wheat was harvested, summer maize was sown. The evident difference of soil moisture from sowing date (orange line) to harvest date (green line) of the summer maize appears to reach a 160 cm soil depth, but is particularly seen within the first 0–120 cm (**Figures 6A–C** and Supplementary Figure S1A). Due to the high rainfall in the growing season of summer maize, the soil water was replenished and recovered. The soil moisture content at the harvest of summer maize had almost recovered by the sowing time of winter wheat under the current irrigation and precipitation regimes (Supplementary Figure S1A).

The variations of soil water content in a 0–180 cm soil depth for winter wheat and summer maize in the SpCSpWS (**Figure 6D**), PWS (**Figure 6E**), and RCPWS (**Figure 6F**) rotations showed similar trends to the WS rotation. Winter wheat depleted the soil moisture from the 0–180 cm soil depth and it fell to its lowest point by the harvest date. It then mostly recovered by the harvest stage of summer maize due to the high precipitation during its growing season (Supplementary Figures S1B–D).

**FIGURE 7:**
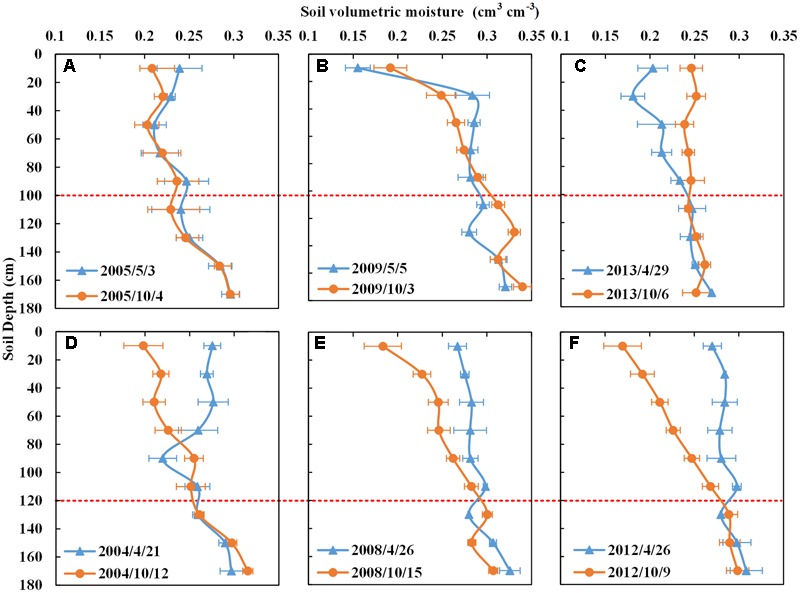
Soil moisture contents with depth of sweet potato and cotton in SpCSpWS rotation for selected years. **(A–C)** Are sweet potato, **(D–F)** for cotton. Blue line represents the soil moisture content with depth at the sowing date; orange line is the moisture content at the harvest date; SpCSpWS rotation, sweet potato→ cotton→ sweet potato → winter wheat-summer maize.

**In the SpCSpWS rotation**, there was no obvious change of soil water content below 100 cm for sweet potato (**Figures 7A–C**). Thus, the soil water depletion of sweet potato occurred in the 0–100 cm soil depth, and particularly in the 0–80 cm interval. In the SpCSpWS cotton rotation years of 2004, 2008, and 2012 (**Figures 7D–F**), an obvious decrease of soil moisture from sowing date to harvest date was present in the 0–120 cm soil depth. Thus cotton was able to use the soil water from 0 to 120 cm, particularly in the 0–100 cm interval, with no notable change below 100 cm. Therefore, shallow rooted sweet potato and cotton could use the upper layer soil moisture, without depleting much at deeper layers, which could later be reached by winter wheat. Much more detail is provided in Supplementary Figure S1D. In the SpCSpWS rotation, winter wheat and summer maize were rotated in 2006, 2010, and 2014 (Supplementary Figure S1D); sweet potato as the preceding crop made use of the soil water in the 0–80 cm depth, which left a favorable soil moisture condition for the succeeding SpCSpWS winter wheat, allowing absorption of moisture from the surface to 180 cm. This rotation received an average of 116 mm per year less irrigation water than the WS rotation.

**FIGURE 8:**
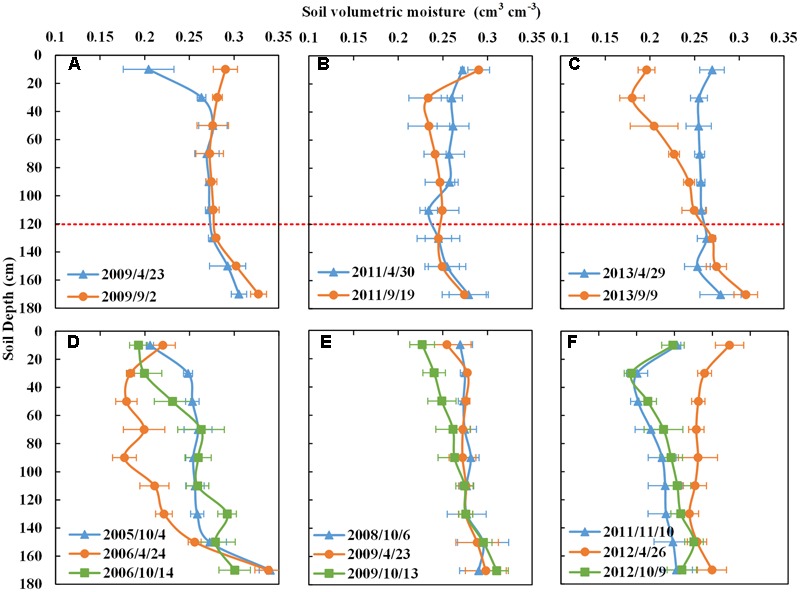
Soil moisture contents with depth of peanut in PWS rotation and ryegrass-cotton in RCPWS rotation in different years. **(A–C)** Are peanut, blue line represents the soil moisture content with depth at the sowing date, orange line is the moisture content at the harvest date. **(D–F)** Are for ryegrass-cotton. Blue line represents the soil moisture with depth at the sowing date of ryegrass; orange line is the moisture content at the harvest date of ryegrass which is the sowing date of the next cotton crop; green line is the mositure content at the harvest date of cotton. RCPWS, ryegrass-cotton→ peanut→ winter wheat-summer maize.

**In the PWS rotation**, obvious changes in soil moisture content of peanut occurred at a 0–80 cm depth from the sowing date to the harvest date (**Figures 8A–C**). Peanuts mainly absorbed soil water from the surface to 80 cm, particularly within the 0–60 cm range. There was no significant difference below the 80 cm soil depth between the harvest and sowing dates of peanut (**Figures 8A–C**). In the year 2009 with a high precipitation of 534 mm and under a consistent irrigation regime of 150 mm, the soil water content of peanut changed most in the 0–40 cm soil layer (**Figure 8A**). The water supply was able to meet the growing requirement of the peanut in 2009. Similarly to sweet potato, peanut crops resulted in only shallow soil moisture depletion, storing the remaining soil water for the succeeding winter of the PWS rotation (more detail is provided in Supplementary Figure S1B). All this while PWS was receiving 90 mm per year less irrigation than the WS rotation.

**In the RCPWS rotation system**, the soil moisture content of ryegrass changed the most in a 0–60 cm soil depth, from the sowing date (blue line) to the harvest date (orange line) (**Figures 8D–F**). Due to the relatively short growing season of ryegrass in winter, and being the cover crop for that season, it mainly consumed the soil moisture of on that the 0–60 cm soil layer. The soil moisture depletion of cotton in the RCPWS rotation is consistent with cotton in the SpCSpWS rotation, mainly focusing on 0–120 cm, and particularly on the first 0–100 cm (**Figures 8D–F**). Thus, ryegrass as the winter cover crop could effectively reduce the non-productive evaporation in the winter fallow season and conserve the soil water. It could also be an effective complement to the soil water uptake by subsequent cotton roots which reach more deeply than ryegrass’s depletion zone of 0–60 cm. However, due to the low rainfall during the ryegrass growing season, 150 mm of irrigation was pumped from the groundwater to ensure growth. Combined water consumptions of the ryegrass and cotton crops within the RCPWS cropping system were higher than that of a one season crop in the SpCSpWS cropping system for the same year (Supplementary Figures S1C,D). However, annual consumption was less than the conventional WS rotation. Similarly, peanut crops could use soil moisture within the 0–80 cm soil depth, particularly from 0 to 60 cm (Supplementary Figure S1C). The soil moisture content below 100 cm was surplus, leaving a favorable soil water condition for the sowing of deeper-rooted winter wheat as the succeeding crop (Supplementary Figure S1C). To sum up, in the RCPWS rotation of ryegrass, cotton, peanuts, winter wheat and summer maize, the diversified crops effectively complemented each other through differences in soil water consumption over various depths. In short, the soil moisture could reach a dynamic balance with the RCPWS rotation of different crops under a total irrigation of 45 mm per year below that used in the WS rotation.

## Discussion

Depletion of groundwater, from using irrigation water to make up for chronic shortfalls in precipitation, is a time limited means to increase crop production. This includes all arid areas with at least one crop and semi-arid areas with two or more crops per year. The Ogallala aquifer in Texas is an example where irrigated crops are replaced by rain-fed crops because the aquifer ran dry. The water crises in the North China Plain is another example where over-exploitation of the ground water resource for crop irrigation to meet the food needs of the 13.9 billion people in China causes a drop of 1 m each year (Fan et al., 2005). We examined here whether cropping systems that alternate shallow and deep rooted crops can save water compared to the traditional rotation of winter wheat and summer maize and how the crop sequence influenced.

### Water Saving for Cropping System Alternating Shallow and Deep Rooted Crops

The results in this study demonstrated that crop rotations involving one shallow rooted crop per year, such as sweet potato and peanut, reduced the average water consumption by 14–28% compared to the conventional WS rotation (**Figure 3**). As our baseline, the average annual evapotranspiration of the WS rotation was 725 mm (**Figure 3**). Liu et al. (2001) observed a longer term range of 800–900 mm *ET_a_* using a large scale weighing lysimeter in which the soil profile was saturated to induce deep percolation, thus allowing *ET_a_* to Zhang et al. (2011) observed 750–850 mm *ET_a_* from 1980 to 2000 s in a 30-year irrigation experiment more in line with our experiments that had be closer to potential by reducing the soil moisture deficit.

Our findings of reduced water consumption for rotations involving shallow rooted crops agree with other studies conducted in the North China Plain (Liu et al., 2008b; Sun et al., 2011; Meng et al., 2012). These studies reported that introducing spring maize into the winter wheat-summer maize rotation lowered annual average *ET_a_* by 12–22% in three sites of the North China Plain from 2004 to 2010. Sun et al. (2015) even asserted that a future shift to alternative crop rotations involving a dryland wheat-maize system, or single wheat or maize is imperative in the water scare North China Plain. Pei et al. (2015) compared the use of ground water between these semi-arid plains and the US High Plains, where water is pumped from the Ogallala aquifer, and recommended switching the current double cropping system to single crop or three crops in 2 years in order to balance *ET_a_* with precipitation and showed that by doing so (as is the case in our experiments) the *ET_a_* and precipitation could be balanced preventing further drawdown of the aquifer and maintain the biomass yield.

### Crop Yield and Water Use Efficiency

Our experiments showed that, by growing a shallow rooted crop the year before a deep rooted crop of winter wheat was planted, the yield of winter wheat and summer maize were improved by 9–16 and 19–32%, respectively, compared to growing continuously a WS rotation (**Figure 4A**). The water use efficiency of winter wheat and summer maize increased by 12–19 and 10–25%, respectively (**Figure 4B**). The improvement in yield is in agreement with the results of Ranamukhaarachchi et al. (2005) and Godfray et al. (2010). These authors rotated wheat, cotton, legume, and sorghum.

Yield improvements in crop rotations involving several crops can result from advantages besides our demonstrated additional water availability, including maintenance of soil productivity and fertility, improved soil structural stability and infiltration capacity (Zhang et al., 2016) and suppression of weeds and diseases by means of resource competition, allelopathic interference, and soil disturbance (Shahzad et al., 2016; Wahbi et al., 2016). Analogous yield improvements to ours were observed in Saskatchewan, Canada, in which durum wheat yield were 7–11% greater following pulse or oilseed crops compared with wheat after spring wheat, attributed in part to greater residual N and soil water prior to the durum wheat planting (Gan et al., 2003).

The increased water use efficiency of winter wheat in our experiment after a shallow rooted crop (**Figure 4B**) was also found by Sun et al. (2011) and Meng et al. (2012) where the *WUE* improved by 5–9%. Our *WUE* of winter wheat and summer maize ranging from 1.73 to 3.06 kg m^-3^ (**Figure 4B**) are greater than the average value of the North China Plain summarized by the review of Fang et al. (2010) which cited a ranged from 1.11 to 2.06 kg m^-3^.

### Soil Moisture Carry Over in Crop Rotations

The soil moisture with depth (**Figures 5–8** and Supplementary Figures S1A–D) helps to explain why winter wheat-summer maize in rotations with shallow rooted crops such as sweet potato and peanut have lower annual average water consumption and greater yield than the continuous WS rotation that receives more irrigation. The roots of sweet potato occur mainly in the 0–100 cm soil zone, which has a strong ability to hold and absorb water above 100 cm soil horizon. The tuberous root of sweet potato is high in moisture content which can provide moisture to the plant in dry years (Ning et al., 2013). The taproot of peanut can be found mainly in 60–90 cm soil depth, and 70% of the gross roots of peanut are located in 0–30 cm (Hong et al., 2009). These shallow rooted crops hardly extract water beyond 120 cm depth as shown in **Figures 7, 8**. The main roots of winter wheat at the maturity stage have been reported to be distributed over 0–200 cm soil depth, with 11% of gross roots deeper than 100 cm (Miao et al., 1989). Therefore, the winter wheat roots could access the water and nutrient below the 120 cm when it is grown in a year following the shallow crops such as in the PWS, RCPWS and SpCSpWS rotations increasing their yield provided that a successful germination occurs (Fang et al., 2010).

Thus alternating shallow and deep rooted crops in the crop rotations effectively can save water in the soil profile from 100 to 200 cm for the deep rooted crop (wheat in this case) from the preceding year when a shallow crop was grown, the soil moisture below 100 cm will not evaporate during the fall and winter when the evaporative demands is low. While our quantitative results are specific to the North China Plain’s semi-arid climate with groundwater irrigation and flat terrain, the findings of complementarity apply well to other area where rainfall and irrigation water are in short supply. The possibility of carrying over wet season recharge in subsoil to support a deeper rooted, dry season crop is worth considering for such other areas.

### Forecast for Water-Saving Farming System in the Future

The present agricultural practices in the North China Plain instituted in the 1980 to overcome food shortages have led to severe environmental degradation and excessive exploitation of water resources. Previous findings (Kendy et al., 2003; Fan et al., 2005; Chen et al., 2011) have shown that improvements in irrigation efficiency are not effective in stopping the decline of ground water in this region. Early in this period large amounts of irrigation water were applied, then water application rates were greatly reduced with irrigation savings practices, yet water table decreased continuously at approximately 1 m per year despite the difference in water application (Kendy et al., 2003; Sun et al., 2015). Only during a year with above normal rainfall were ground water declines under 1 m per year. The reason for the insensitivity to irrigation efficiencies can be explained by a simple mass balance in which the water loss of the soil profile including the ground water is the difference between the precipitation and actual evaporation. All excess irrigation water flows back to the groundwater and is thus not a net loss in this system. Thus, during a high rainfall year, the difference between evaporation and precipitation is smaller and hence the groundwater storage decreases less.

Consequently, the most important way to increase the water use efficiency is either to save water by reducing the evaporation or to produce higher yield with the same evaporation. The proposed rotations in this paper in which sallow rooted crops and deep rooted crop are alternated, increase the water use efficiency of winter wheat. Therefore, these rotations are more beneficial than increasing irrigation efficiency.

Evaporation can be even further reduced for the proposed rotations by introducing water-saving technologies that reduce evaporation from the soil (e.g., mulching, drip irrigation, and regulated deficit irrigation in combination with the use of crop varieties resistant to drought and heat) and agronomic measures such as conservation tillage. Yield can be increased by recycling of organic manures, or by incorporating more effective fertilization by integration of water and fertilizer application.

When all of these aspects are integrated and tested successfully, it is possible to reduce the decline of the groundwater during dry years and perhaps increase groundwater tables during wet years. In short, this study provides an additional tool for policy-makers and government to fashion new cropping systems that are resource-conserving, environment-friendly, and climate-smart, yet still meet food production demands in an extremely water scarce region, the North China Plain.

## Conclusion

Water-wise alternative crop rotations with shallow- and deep- root crop alternation produced effective soil moisture complementarity through rotation sequencing, via moisture carryover in the deeper vertical profile. By introducing an annual crop such as cotton, peanut and sweet potato preceding winter wheat, alternative crop rotations could effectively reduce their annual average water consumption and improve the annual yield, both contributing to improved water use efficiency in winter wheat compared to the conventional winter wheat-summer maize rotation. Therefore, alternative crop rotation is a promising option for maintaining food production and alleviating the groundwater table decline in the North China Plain. It provides useful information for meeting food production demands and decreasing the over exploitation of groundwater in an extremely water scarce region, the North China Plain in other, similar in severity, water-shortage regions.

## Author Contributions

X-LY, Y-QC, PS, and W-SG designed research; X-LY, LM, MZ, and PS performed research; X-LY, SP, and TS analyzed data; and X-LY, Y-QC, and TS wrote the paper. All authors discussed the results.

## Conflict of Interest Statement

The authors declare that the research was conducted in the absence of any commercial or financial relationships that could be construed as a potential conflict of interest.

## Abbreviations

C: cotton
P: peanuts
PWS: peanuts → winter wheat-summer maize
R: ryegrass
RCPWS: ryegrass–cotton→ peanuts→ winter wheat-summer maize
S: summer maize
Sp: sweet potato
S_P_CS_P_WS: sweet potato→ cotton→ sweet potato→ winter wheat-summer maize
W: winter wheat
WS: winter wheat-summer maize
→: fallow gap between crops
-: no fallow gap between crops
