# Supplementary material for: Mitigating Groundwater Depletion in North China Plain with Cropping System that Alternate Deep and Shallow Rooted Crops
Source: Front Plant Sci. 2017 Jun 8;8:980. doi: 10.3389/fpls.2017.00980 (PMC5463059; doi:10.3389/fpls.2017.00980)
Supplement: Supplementary file 1 [file Table_1.docx]

***Supplementary Material***

**Mitigating groundwater depletion in North China Plain with cropping system that alternate deep and shallow rooted crops**

Xiao-Lin Yang^1‡^, Yuan-Quan Chen^1‡^, Tammo, S. Steenhuis^2^, Steven Pacenka^2^, Wang-Sheng Gao^1^, Li Ma^1^, Min Zhang^1^, Peng Sui^1*^

^1^College of Agronomy and Biotechnology, China Agricultural University, Beijing 100193, People’s Republic of China

^2^Department of Biological and Environmental Engineering, Riley-Robb Hall, Cornell University, Ithaca, NY14853, United States of America

^‡^ The authors contributed equally to this work.

^*^ Corresponding authors: Peng Sui, phone: +86 10 62731436; Fax: +86 10 62731436; E-mail: [suipeng@cau.edu.cn](mailto:suipeng@cau.edu.cn)

1. **Figure S1 legend**

**Figure S1** Soil moisture contents with depth of the 0-180 cm soil profile of each crop, each year in four crop rotations from 2003 to 2014. (A: WS rotation (winter wheat-summer maize); B: PWS rotation (peanut→winter wheat-summer maize); C: RCPWS (Ryegrass-cotton→peanuts→winter wheat-summer maize); D: SpCSpWS rotation ( sweet potato→cotton→sweet potato→winter wheat-summer maize); The small letters from a to i are the soil moisture content of each crop at each year. In the small Figs with two lines, blue line represents the sowing date; orange line is the harvest date. In the small Figs with three lines: blue line represents the sowing date of winter wheat; orange line means the harvest date of winter wheat (it is also the sowing date of summer maize). The green line means the harvest date of summer maize.

1. **Supplementary Tables**

**Table S1** Experiment management of each crop

|  | Winter wheat | Summer maize | Sweet potato | Cotton | Peanut | Ryegrass |
| --- | --- | --- | --- | --- | --- | --- |
| Total irrigation amount (mm) | 225 | 105 | 150 | 225 | 150 | 150 |
| Irrigation times | Sowing/jointing/filling | Jointing/filling | Sowing/middle | Sowing/squaring/boll opening | Sowing/flowering/podding | Sowing/turning green |
| N (kg/ha) | 225 | 180 | 70 | 70 | 75 | 225 |
| P_2_O_5_ (kg/ha) | 112.5 | 105 | 97 | 97 | 60 | 112.5 |
| K_2_O (kg/ha) | 225 | 75 | 128 | 128 | 135 | 225 |
| Planting date | 6 Oct | 16 Jun | 30 Apr | 23 Apr | 23 Apr | 4 Oct |
| Harvest date | 13 Jun | 2 Oct | 2 Oct | 4 Oct | 26 Aug | 22 Apr |
