# Supplementary material for: Mitigating Groundwater Depletion in North China Plain with Cropping System that Alternate Deep and Shallow Rooted Crops
Source: Front Plant Sci. 2017 Jun 8;8:980. doi: 10.3389/fpls.2017.00980 (PMC5463059; doi:10.3389/fpls.2017.00980)
Supplement: Supplementary file 2 [file Table_2.DOCX]

**Table S2** Annual actual evapotranspiration (*ET_a_*) of four crop rotations from 2003-2014 (mm)

| Year | SpCSpWS | WS | PWS | RCPWS |
| --- | --- | --- | --- | --- |
| 2003 | 522±11d | 722±3a | 525±1d | 703±3b |
| 2004 | 637±1b | 808±12a | 801±9a | 550±25c |
| 2005 | 555±25bc | 793±12a | 520±32c | 782±12a |
| 2006 | 724±1a | 695±1b | 729±7a | 681±3c |
| 2007 | 436±8d | 641±2a | 451±11c | 443±4cd |
| 2008 | 547±3c | 746±3b | 751±1b | 831±8a |
| 2009 | 509±2e | 758±1a | 546±3d | 740±2b |
| 2010 | 696±12a | 701±9a | 689±6a | 471±28b |
| 2011 | 437±35b | 713±16a | 467±14b | 691±16a |
| 2012 | 532±5d | 732±4b | 751±9a | 696±12c |
| 2013 | 519±5d | 769±4a | 531±6c | 562±3b |
| 2014 | 614±21a | 616±5a | 621±24a | 621±5a |
| average | 561d | 725a | 615c | 647b |

Note: Different lowercase letters after the values in the same row demonstrated significant difference at the 0.05 level. ± donate Standard Deviation. SpCSpWS rotation: sweet potato→ cotton→ sweet potato→ winter wheat-summer maize; RCPWS: ryegrass-cotton→ peanut→ winter wheat-summer maize; PWS: peanut→ winter wheat-summer maize; WS: winter wheat-summer maize.
