# Supplementary material for: Mitigating Groundwater Depletion in North China Plain with Cropping System that Alternate Deep and Shallow Rooted Crops
Source: Front Plant Sci. 2017 Jun 8;8:980. doi: 10.3389/fpls.2017.00980 (PMC5463059; doi:10.3389/fpls.2017.00980)
Supplement: Supplementary file 3 [file Table_3.DOCX]

**Table S3** Comparison of soil moisture storage changes of 0-180 cm soil profile between SpCSpWS rotation and WS rotation at each rotation cycle from 2003 to 2014 (mm)

| Period | WS  rotation | SpCSpWS rotation |
| --- | --- | --- |
| within the first crop rotation：2003/3/30-2005/10/4 | -16 | 47 |
| within the second crop rotation：2006/10/4-2009/10/3 | 44 | 84 |
| within the third crop rotation：2010/10/7-2013/10/6 | -51 | 55 |

Note：The period in the first column is from the beginning to the sowing date of winter wheat in each rotation cycle. Values in the second and third column are the changes of soil moisture storage content of the 0-180 cm soil profile. Positive values indicate that the soil water storage increased, negative values indicate that the soil water storage decreased. SpCSpWS rotation: sweet potato→ cotton→ sweet potato→ winter wheat-summer maize; WS: winter wheat-summer maize.
