# Supplementary material for: Mitigating Groundwater Depletion in North China Plain with Cropping System that Alternate Deep and Shallow Rooted Crops
Source: Front Plant Sci. 2017 Jun 8;8:980. doi: 10.3389/fpls.2017.00980 (PMC5463059; doi:10.3389/fpls.2017.00980)
Supplement: Supplementary file 5 [file Table_5.docx]

**Table S5** Comparison of soil moisture storage changes of 0-180 cm soil profile between RCPWS rotation and WS rotation at each rotation cycle from 2003 to 2014 (mm)

| Period | WS  rotation | RCPWS rotation |
| --- | --- | --- |
| Within the first rotation cycle：2003/3/30-2004/10/2 | 24 | 114 |
| Within the second rotation cycle：2005/10/4-2007/10/8 | 64 | 35 |
| Within the third rotation cycle：2008/10/15-2010/10/17 | -34 | -43 |
| Within the fourth rotation cycle：2011/9/27-2013/10/6 | -28 | 26 |

Note: The period in the first column is from the beginning to the sowing date of winter wheat in each rotation cycle. Values in the second and third column are the changes of soil moisture storage content of 0-180 cm soil profile. Positive values indicate that the soil water storage increased, negative values indicate that the soil water storage decreased. RCPWS: ryegrass-cotton→ peanut→ winter wheat-summer maize; WS: winter wheat-summer maize.
