# Supplementary figures and images for: Mitigating Groundwater Depletion in North China Plain with Cropping System that Alternate Deep and Shallow Rooted Crops
Source: Front Plant Sci. 2017 Jun 8;8:980. doi: 10.3389/fpls.2017.00980 (PMC5463059; doi:10.3389/fpls.2017.00980)

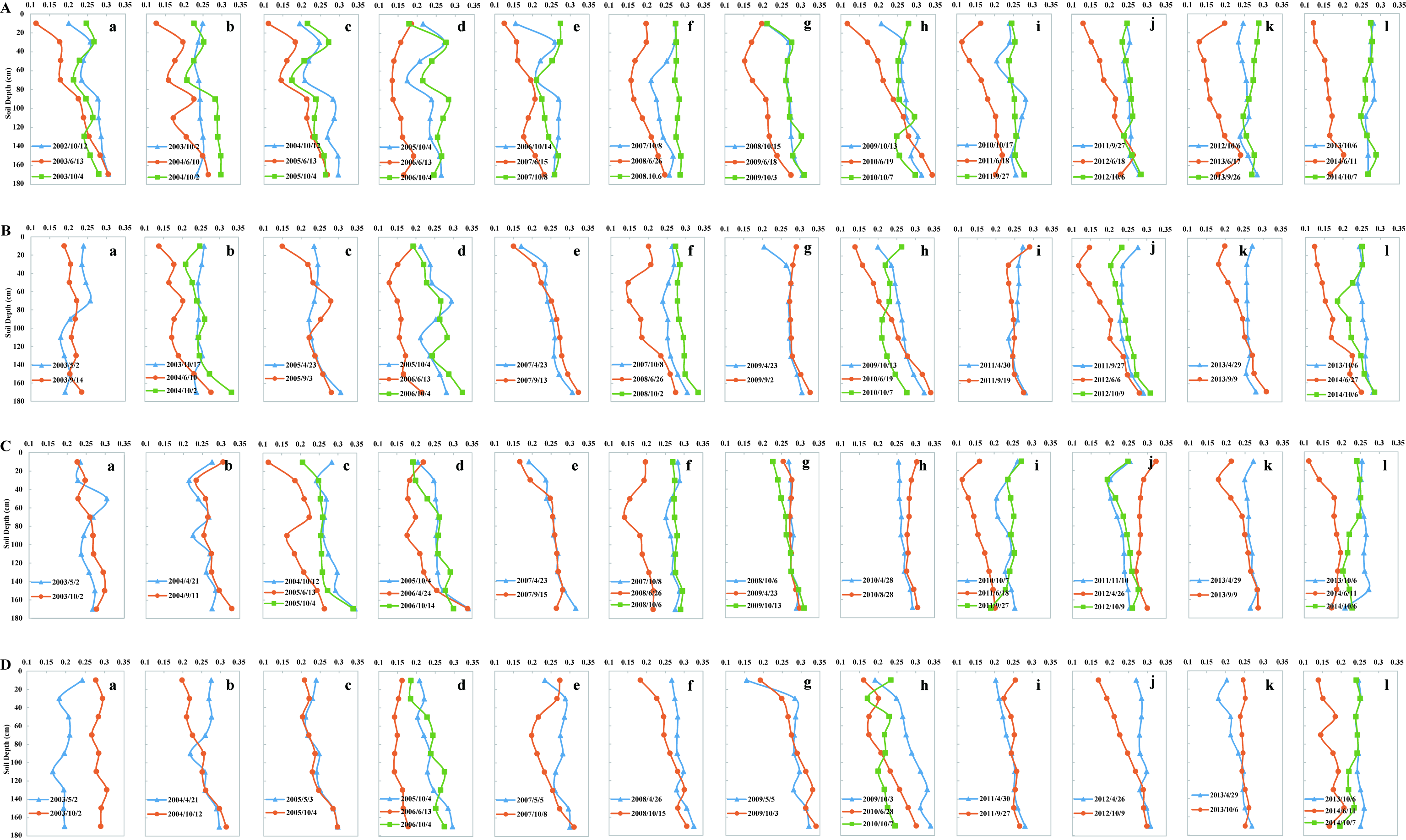

Supplement: Supplementary file 6 [file Image_1.TIF]
